# Mechanisms of Activation of Voltage-Gated Potassium Channels

**Published:** 2014

**Authors:** A. V. Grizel, G. S. Glukhov, O. S. Sokolova

**Affiliations:** Saint Petersburg State University, 7-9, Universitetskaya nab., 199034, St. Petersburg, Russia; Biological Faculty of Moscow State MV Lomonosov University, 1, Leninskie Gory, Bld. 12, 119991, Moscow, Russia

**Keywords:** activation, potassium ion channels, modeling, structure

## Abstract

Voltage-gated potassium ion channels (Kv) play an important role in a variety of cellular processes, including the functioning of excitable cells, regulation of apoptosis, cell growth and differentiation, the release of neurotransmitters and hormones, maintenance of cardiac activity, etc. Failure in the functioning of Kv channels leads to severe genetic disorders and the development of tumors, including malignant ones. Understanding the mechanisms underlying Kv channels functioning is a key factor in determining the cause of the diseases associated with mutations in the channels, and in the search for new drugs. The mechanism of activation of the channels is a topic of ongoing debate, and a consensus on the issue has not yet been reached. This review discusses the key stages in studying the mechanisms of functioning of Kv channels and describes the basic models of their activation known to date.

## INTRODUCTION

**Fig. 1:**
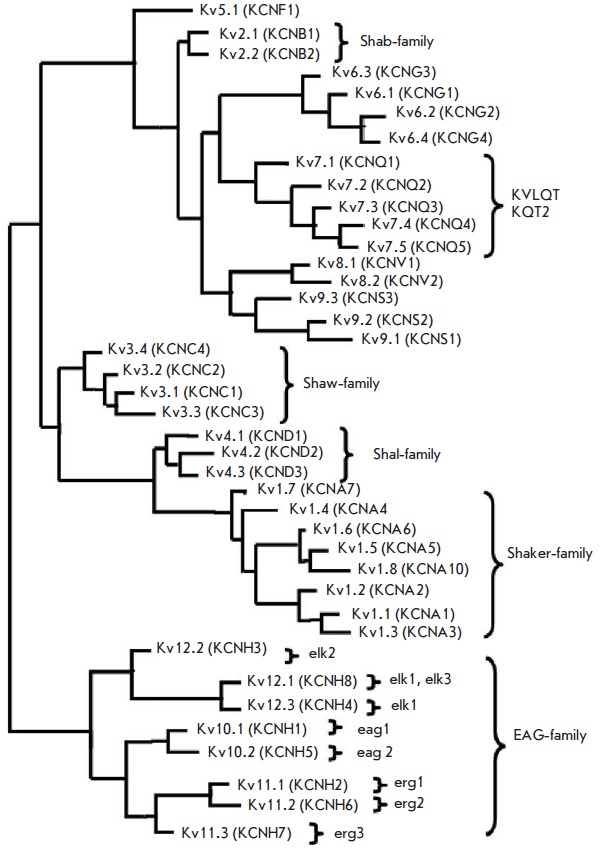
The phylogenetic tree of Kv channels based on the alignment of amino acid sequences. Braces combine channels belonging to the same family. Names are given according to the system of the International Union of Pure and Applied Chemistry (IUPAC) (alternative names according to the Gene Nomenclature Committee of the Human Genome Organization [2] are given in brackets)

Membrane proteins account for ~30% of the total proteome of an organism, with about half of this number being carrier proteins and ion channels. Potassium ion channels represent the most diverse and widespread class of membrane proteins [1]. Depending on the functioning principle and based on the primary structure of a channel-forming subunit, these proteins are subdivided into inwardly rectifying channels (Kir), Ca^2+^-activated channels (KCa), two-pore domain (K2P), and voltage-gated (Kv) channels. Kv channels form the most diverse group (*Fig. 1*), represented by 12 families (Kv1-Kv12) [2].

All Kv channels play an important regulatory role in various cellular processes. These proteins participate in the functioning of excitable cells [3-5], regulation of apoptosis [6], as well as cell differentiation and growth [7]. Correct functioning of Kv channels is necessary for the release of neurotransmitters [8] and hormones [9, 10], for maintenance of cardiac activity [11], etc.

Mutations in the genes of Kv channels can lead to various severe hereditary disorders [12], including deafness, epilepsy [13], and certain types of cardiac rhythm disorders [11]. They are also involved in the pathogenesis of multiple sclerosis and the pain syndrome [14]. Kv channels are also linked to the processes of tumor onset and development, including in malignant tumors [15].

Functioning of Kv channels can be modulated by using activators and blockers [16]; therefore, they represent perspective drug targets [17, 18]: hence, studying the structure and function of Kv channels is an important task.

## QUATERNARY STRUCTURE OF Kv CHANNELS

**Fig. 2:**
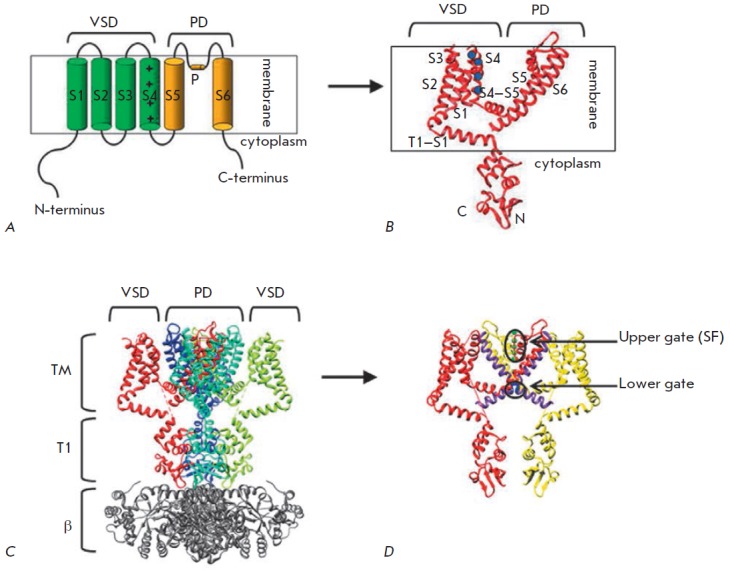
Structure of Kv channels. A. Scheme of a single α-subunit of the Kv channel. Transmembrane segments S1–S6 and pore-forming P-loop are marked. Charged Arg of the membrane voltage sensor S4 are marked with “+” signs. PD –pore domain. B. Crystal structure of a single α-subunit of the Kv1.2 channel [21]. S1–6 segments, cytoplasmic domain T1, linker connecting the transmembrane portion with the T1 domain (T1–1), as well as N- and C-termini are marked. Charged Arg residues of the membrane voltage sensor S4 are indicated by blue circles. C. Crystal structure of the Kv1.2 channel in a complex with the β-subunit (marked as β, grey colored) (modified from [21]). TM –transmembrane region. D. Gate of the Kv2.1 channel. Only two opposite subunits of Kvα are shown for clarity reasons. The S6 helix is shown in purple, the blue color denotes a highly conserved portion of S6T –PXP helix (Pro-Val-Pro in Kv2.1), a key component of the lower gate. Green spheres mark K^+^ ions in the selectivity filter (P-loop), which represents the upper gate of the channel

All Kv channels have a high level of similarity. Each Kv channel gene encodes one α-subunit (Kvα). Four α-subunits are required to form a functional channel (*Fig. 2*) [19, 20]. Kv channels usually have a homotetrameric structure (with all Kvα being identical) [19, 20]; however, some channels can be heterotetrameric (with two or more non-identical Kvα subunits).

The transmembrane domain of the Kv channel α-subunit consists of six helices: S1–S6 (*Fig. 2A,B*). These helices form two structurally and functionally different parts of the tetrameric channel: 1) a potassium ion-conducting domain (pore domain) – helices S5–S6 located in the channel center, and 2) a domain sensible to changes in the membrane potential (voltage-sensing domain, VSD) – helices S1–S4 located on the channel periphery (*Fig. 2B,C*).

The pore part includes a channel gate and a selective filter that does not allow ions other than K^+^ to penetrate through the channel. The channel gate is formed by crossing C-termini of the S6 helices that block passage of ions when the channel is closed [22-24]. A conserved fragment (P-region) and a S5–S6 loop participate in the formation of the selectivity filter of the channel (*Fig. 2*).

It is known that VSD and the pore domain are covalently bound by the S4–S5 linker, an amphiphylic helix connected to the C-terminus of S6 helix (S6T) and the next subunit [21, 25-30]. The highly conserved region of the S6T helix plays an important role in the opening/ closing of the channel gates and consists of two Pro residues usually separated by Val or another amino acid, PXP (*Fig. 2D*). This region is flexible, which allows the channel to open [21]. Kv channels have two gates: (1) the lower gate (LG) formed by crossing the S6 helices on the intracellular side, and (2) the upper gate (UG) formed by the P-loop of the selectivity filter on the extracellular side (*Fig. 2D*). In the Kv channels, as well as in the majority of other potassium channels, LG are the main activation gates controlled by external stimuli, such as the membrane potential. Inner S6 helices intercross, similarly to the blades in the iris diaphragm of a photographic camera, and they open/close in a similar manner.

Besides the transmembrane part, Kv channels have a cytoplasmic part formed by N- and C-termini (*Fig. 2*). The cytoplasmic part does not contain highly conserved regions and is different for Kv channels from different families [31].

In cells, Kv channels function in the form of large macromolecular complexes comprising ion-conducting α-subunits, auxiliary cytoplasmic and/or transmembrane β-subunits, as well as regulatory and supporting proteins [32] (*Fig. 2C*). The assembly of pore-forming α-subunits and the auxiliary subunits of Kv channels in mammals takes place in the endoplasmic reticulum, where they form a stable complex [33]. α-Subunits form the ion pore, while β-subunits (*Fig. 2B,C*) and other auxiliary subunits modulate the properties and functions of α-subunits. This complexity of the structural forms determines the wide variety of the properties and functions of Kv channels [34].

## ACTIVATION OF Kv CHANNELS

**Fig. 3:**
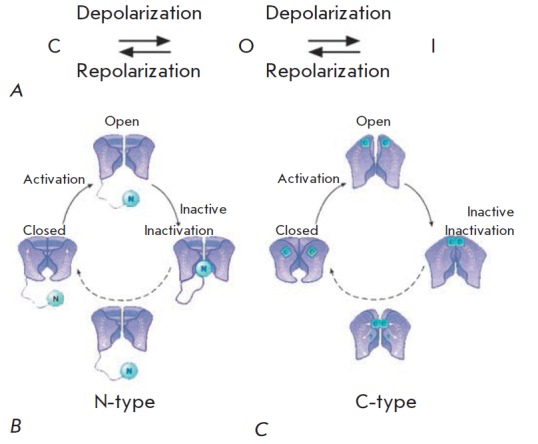
A. Scheme of the conformational transitions in Kv channels: C – closed channel; O – open channel; I – inactivated channel. B. N-type inactivation. The inactivation peptide enters the pore and physically blocks the transfer of ions after the activation of the channel. C. C-type inactivation. The selectivity filter acts as the second gate and closes, preventing the penetration of ions. The channels completely return to the closed conformation when the potential drops to the resting potential level (modified from [35])

All Kv channels share a similar mechanism of activation. They can be present in three functional states: quiescent state (closed conformation) ↔ activated state (open conformation) ↔ inactivated state (*Fig. 3*).

The channel does not conduct the ions in the quiescent state. Depolarization of the membrane results in positive charge of its intracellular part, causing conformational rearrangements of Kv channels and making an open conformation energetically favorable. This rearrangement is termed the activation of channel [36]. In case the membrane remains depolarized, the majority of Kv channels switch to the inactivated non-conducting state. Two basic inactivation types termed N and C have been described so far (*Fig. 3*). Fast N-type inactivation is mediated by an inactivation peptide folded into a globule and attached by a linker to the N-terminus of either an α-subunit (α-ball) or a β-subunit (β-ball) [3, 37]. The inactivation peptide enters the open pore of the channel and blocks ion traffic [3, 38, 39]. In case of slow inactivation (C-type), the selectivity filter acts as the second gate and closes, preventing the entry of ions [4, 40-42]. The channels completely return to the closed conformation after the inactivation when the potential drops to the resting potential level.

The mechanism of channel activation remains a topic of debate. Knowledge of the atomic structure of the channel in various functional states and in at least two final conformations (open and closed) is necessary for a comprehensive understanding of this issue. The majority of the crystal structures of Kv channels [21, 43, 44] have been obtained in the open state; therefore, the models of Kv channels activation are often created on the basis of structural information acquired by various experimental approaches and molecular modeling (MM) [43, 45-53]. These models form the basis for reasonable interpretations of the obtained results and design of further experiments. As of today, an extensive amount of data pointing to the features of the Kv channels structure in the open and closed conformations has been collected. The modern models of activation largely converge to a single consensus model of channel opening [54-57]. All these models are based on earlier key experiments and activation models.

## EXPERIMENTAL DATA FOR THE MODELING OF ACTIVATION OF Kv CHANNELS

It was suggested in the very first models of Kv channels activation that the change in the transmembrane potential during the activation of the channel caused the voltage sensor S4 to move upstream of the channel that was connected to the external and internal solutions [58]. Later on, experimental data on the functioning of Kv channels were accumulated, allowing one to refine the available models of activation. The fundamental data important for deciphering the activation mechanism of Kv channels are as follows:

(1) *S4 segment contains a conserved repeated motif of three amino acid residues: (+, X1, X2, +, X1, X2 …).*

Mutational and electrophysiological analyses allowed researchers to identify the most significant (HI – high-impact) and least significant (LI – low-impact) residues for the process of channel activation/deactivation [59]. The voltage sensor S4 contains the conserved sequence (+, X1, X2, +, X1, X2 …), where (+) is a positively charged HI (significant) residue, (X1) is a hydrophobic HI residue, and (X2) is a hydrophobic LI (non-significant) residue (*Fig. 3A*) [60, 61]. X1 residues are located in a protein environment where their mutations may lead to a disruption in protein folding and, consequently, to disruption in the channel opening/ closing process. X2 residues are exposed in the lipid or water environment, and their impact on channel functioning is insignificant. The repeat (+, X1, X2, +, X1, X2 …) forms three parallel left-handed coils with a small inclination along the right-handed S4 helix.

(2) *Each subunit possesses approximately three gate charges located on the R1–R4 residues of the S4 helix.*

The movement of the voltage sensor S4 can be detected by measuring the gate currents resulting from the movement of the electrostatic charges of the S4 helix relative to the electric field. The transition of the* Shaker *channel from the quiescent to the activated state is accompanied by the transfer of ~3.2–3.4 charges per subunit [62-64]. The method of alternate neutralization of the negative charges of S2/S3 helices and the positive charges of S4 allowed researchers to identify the amino acid residues responsible for the transfer of the gate charge [63, 64] as R1, R2, R3 and R4 [65, 66].

(3) *10 amino acid residues of the S4 segment are located in the membrane.*

Substitution of certain amino acid residues of the* Shaker *channel for Cys showed that the sequence of ~10 aa is inaccessible to both intracellular and extracellular solvents while the channel remains in the quiescence state [67, 68]. This sequence corresponds to ~13.5 A of the α-helix and can include only two or three positive charges of S4 (*Fig. 3B,C*). Accordingly, there are deep water antechambers on both sides of the membrane and only a small portion of S4 is located in a short GC (gate channel) (*Fig. 3B,C*).

(4) *The S4 helix is able to move in a water-filled cleft called gate channel (GC) with a very narrow barrier between the external and internal solutions.*

The three “sides” of the GC are formed by S2/S3 helices, the pore domain, and lipids. An interaction between three conserved negative amino acid residues in the S2 and S3 helices and the positive residues in S4 indicates that S2 and S3 are located on one side of the GC [63, 69-71].

According to fluorescent and mutational analysis data, the pore domain is located on the other side of GC [72, 73]. This is supported by the fact that the R1 and R2 residues of the S4 helix approach E418 of the pore domain during activation [74, 75], while Cys inserted in S4 can form a bond with Cys introduced in the pore domain [45, 59].

The third side of GC is apparently formed by lipids, corresponding to the hydrophobic nature of a residue of the S4 helix at the X2 position. A weak relationship between the mutations in these residues and channel activation also points to this fact [60, 61].

(5) *Activation leads to a shift of the S4 segment by 9 amino acid residues.*

The fluorescence measurement method shows that the activation process is connected to the movement of ~9 aa of S4 from GC into the external solution [61, 67, 68, 76, 77]. At the same time, the sequence of ~9 aa disappears from the inner water antechamber [67, 68]. (6) *The S4 helix rotates during activation.*

It has been established using the FRET method that S4 rotates by ~180o during the activation of the channel [78, 79].

(7) *The membrane voltage sensor S4 has a stable intermediate state.*

Two phases in the movement of gate charges [80] and two consecutive movements of gate charges in the external direction with an intermediate transmembrane position of S4 [68] were established by kinetic studies.

(8) *The channel can form a proton pore.*

A substitution of R1 or R4 for His allows the channel to conduct protons (omega-current) [65, 66]. Consequently, the channel may contain a water channel that serves as a bridge between the internal and external solutions, while R1H forms the proton pore in the quiescent state and R4H – in the activated state.

## FUNDAMENTAL MODELS OF ACTIVATION OF Kv CHANNELS

**Fig. 4:**
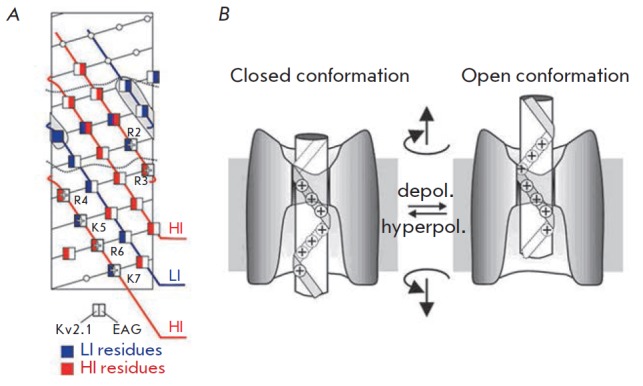
A. Schematic arrangement of S4 helix residues in the Kv10 and Kv2.1 channels. The distribution of residues with high impact (HI) and low impact (LI) on the opening/closing process is shown. Three parallel stripes along the S4 helix (HI charged residues, HI hydrophobic residues, LI hydrophobic residues) are continuous for both channels and form a three-step coil [59]. B. Kv channel activation scheme according to the SHM model [59] – screw rotation and motion of S4 helix (white cylinder) in a fixed gate channel (GC)

(1) A sliding-helix model (SHM) [59] was proposed basing on the key facts reviewed above. The pore domain in a model of the Kv channel was reconstructed on the basis of the structure of the homologous potassium channel KcsA, while the location of VSD helices remained unknown at that time. The SHM model describes in details only the relative position of some amino acid residues of S1-S4 helices. According to this model, the rather short (~13.5 A) GC channel has large antechambers filled with water on both the outer and inner sides. Due to this space, the electric field focuses on the small S4 portion, minimizing contact between several charged amino acids and the dielectric environment and providing a large gate charge. The activation is accompanied by a screw motion of S4 perpendicularly to the membrane surface in three separate steps. The screw motion during the activation can be stopped in stable intermediated states, in which the basic charges (R1-R4) move to the position occupied by the previous charge (*Fig. 4A*). The positively charged R1-R4 residues form consecutive ionic pairs with the negatively charged amino acid residues of neighbor helices. Each step is accompanied by the transfer of 1/3 of the total charge (~3) per subunit; i.e., 1 charge in general (*Fig. 4*) [59].

(2) *A paddle model (PM) *is a completely different activation model that emerged after the deciphering of the crystal structure of the bacterial channel KvAP [43, 81].

S3-S4 helices in the crystal structure of the KvAP channel are located close to the intracellular surface of the membrane and perpendicular to the pore axis (*Fig. 4B*). It has been shown that the S3 helix consists of two fragments (S3a and S3b) connected by the S3 loop (*Fig. 3B*). The S3b segment and N-terminal part of the S4 helix are oriented in antiparallel strictly opposite each other, forming a hydrophobic element with an helix-loop-helix structure that is attached to the pore domain via the flexible loop of the S3 helix and S4-S5 linker (*Fig. 3B*). This S3b-S4 element was termed the “paddle” [43], giving its name to the concerned model.

According to the PM, the positively charged paddles of the channel in closed conformation are located near the intracellular surface of the membrane; they are held in this position by a large electrical field, while the membrane resting potential is negative. In response to depolarization, the paddles move simultaneously through the membrane towards the external side, pulling along the S4-S5 linker that in turn pulls the S5 helix away from the pore axis.

This model conforms to some experimental data [46, 81] that point to a possible long-distance movement of a paddle of the potential sensor (about 20 A). However, later experiments have shown that the used crystal structure of KvAP [43] possesses a non-native conformation [82].

Acquisition of new structural data [65, 67-69, 71, 73, 83-86] allowed researchers to propose *(3) an advanced SHM model *[87]. It was created on the basis of the data on the sequence of the *Shaker *channel and the crystal structure of the KvAP channel [43]. Similar to the previous model [59, 88], the new model suggests that the movement between the open and closed conformations of the channel includes three consecutive screw motions, when S4 moves by ~13.5 A along the axis and rotates by 180o. At the same time, the positively charged S4 groups remain in the polar environment where they can interact with the negatively charged residues of S1-S3 helices, with other polar atoms, negatively charged lipid heads, and water. There is a single barrier dividing the amino acid residues of S4 into ones accessible from outside and inside (*Fig. 4B*) in this model. It provides a more detailed description of the interaction of the different amino acids of VSD with each other and includes a modeling of the channel pore part.

**Fig. 5:**
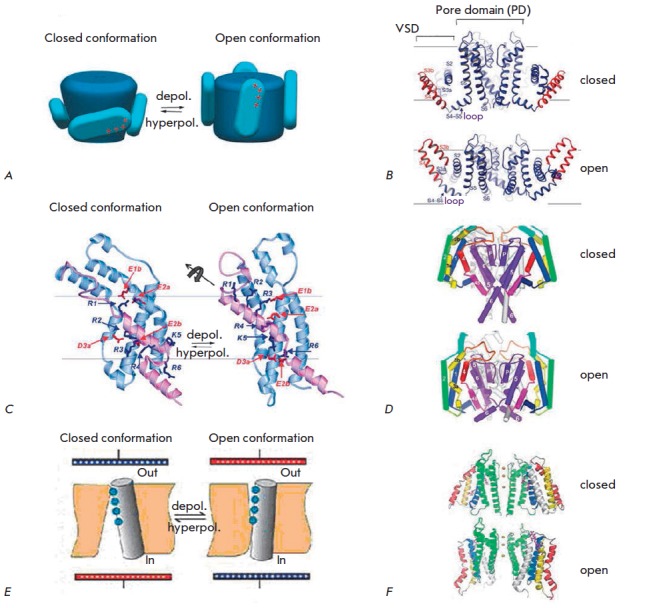
Various models of Kv channels activation. All channels and their parts are shown in lateral orientation: the extracellular space is at the top, and the cytoplasm is at the bottom. A. Scheme of the paddle model (PM) of Kv activation. The movement of the paddles (blue ovals) is shown. Red “+” signs mark Arg in the S4 helix. B. PM based on the crystal structure of KvAP [92] in open and closed conformation. Paddle S3b–4 is shown in red. S1–4 helices are marked. The channel is shown as a frontal section. C. Sliding-helix model (SHM). The changes in the VSD domain of the *Shaker* channel are shown. Movable S4 segments and the S4–5 loop are purple-colored. Positively charged side chains of the S4 helix and negatively charged side chains of the S1–3 helices interacting with each other are colored blue and red, respectively [83]. D. SHM. The full-sized channels are given in closed and open conformations. S1–6 helices are numbered. Helices of the VSD domain are shown in different colors. Helices of the pore domain (S5–6) are purple-colored [83] E. Scheme of S4 helix movement (grey cylinder) during the activation of the Kv channel, according to the transport model (TM), showing how depolarization changes the availability of Arg residues (shown as blue circles) from the inner and outer aqueous cavities [48]. F. TM of Kv channels activation –closed and open conformations of the *Shaker *channel are shown. Transmembrane helices are color-coded: S1 –white, S2 –yellow, S3 –red, S4 –blue; pore domain is shown in green; Arg in S4 are shown in purple [48]

Later data of FRET [78] and potentiometric studies [89] showed that S4 virtually does not move towards the transmembrane direction [48], which contradicts both SHM and, especially, PM. Moreover, in the open state, the upper part of the S4 sensor interacts with the pore domain, which is impossible according to PM. Those data as well as earlier studies [45, 84, 89, 90] gave rise to *(4) a transport model (TM) *of activation [48, 84, 91] (*Fig. 5A,B*).

According to the TM model, similar to the SHM, the channel has deep water cavities on both sides of the membrane, divided by a small area of the channel in the middle of the membrane; the electric field focuses exactly on this spot, and thus the transfer of gate charges from one side of the membrane to another does not require big S4 movements. S4 changes its position during channel activation, tilting by 45o, but at the same time moving perpendicularly to the membrane surface to a small distance (less than 2 A), while Arg on this helix move from the deep aqueous cavity on the intracellular side to the cavity on the outer side of the membrane. This relocation of Arg is possible due to two barriers that control the accessibility of S4 amino acid residues to water from the inner and outer sides (*Fig. 5*). The movement of the S4 helix combines rotation and inclination, and the helix always stays in the polar environment (*Fig. 5A,B*). In the TM model, S4 may be qualitatively compared to a transporter that has its binding site accessibility changing between the inner and outer sides in each cycle. This evolutionarily conserved mechanism is sufficient for the transfer of a large amount of charge through the electric field without movement of S4 through the membrane. The TM model conforms to much of the experimental data [47, 60, 72, 73, 78, 89, 93].

The biggest difference between the fundamental models consists in the amplitude of the S4 segment movement, which may be a consequence of the simplifications adopted in these models. For example, the movement of S4 in the SHM model is basically represented as the motion of a rigid body; however, it has been shown that S4 can transit from the α-helix conformation to a 310 helix [44, 54, 94-97]. The PM model assumes that the S3-S4 paddle moves as a single entity, but experiments demonstrate that these two helices move independently [98].

## MODERN MODELS OF Kv CHANNELS ACTIVATION

New data on the open conformation of the Kv channel became available after the crystal structures of the eukaryotic channel Kv1.2 (*Fig. 2C*) [21] and Kv1.2/Kv2.1 chimera [44] were determined, with the first one being improved later on [99]. The advances in computer software and methods allowed researchers to compute more complicated molecular models and to study the functioning mechanisms by means of molecular dynamics. This led to the emergence of several new models and hypotheses regarding Kv channel activation, including *(5) a model of coordinated movement of helices (CMH) *[53].

A molecular model of the eukaryotic channel Kv2.1 in the closed state and the CMH model of activation for this channel were created using the data of an X-ray structural analysis (*Fig. 2C*) [53]. *De novo *modeling (Rosetta method), the molecular dynamics method (MD), and voltage-clamp fluorometry (VCF) data were used for that purpose.

**Fig. 6:**
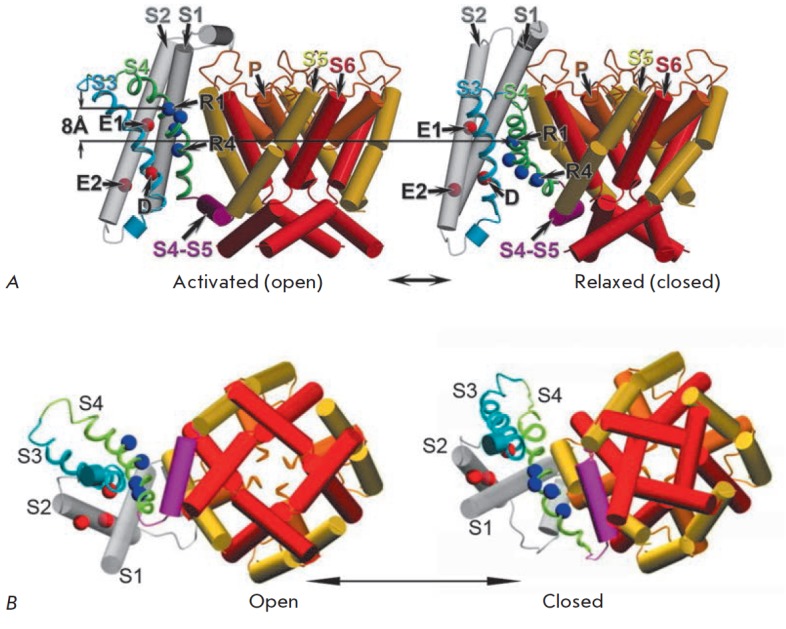
Comparison of Kv1.2 channel models [53] in the activated (open) state (left) and in the rested (closed) state (right). All transmembrane helices are shown as cylinders, except for S3 and S4 shown as spirals. Only one VSD domain is shown. S1 and S2 helices are shown in grey; the S4– S5 linker is purple-colored. The positions of Cα carbon atoms of Arg in the S4 helix are marked as R1 and R4 and highlighted in blue. The amino acid residue E226 of the S2 helix is marked as E1; E236 of the S2 helix, as E2; and residue D259 of the S3 helix, as D; these amino acids are highlighted in red. A. Lateral view. B. View from the extracellular space

According to the CMH model, during depolarization of the membrane, S4 moves as an inclining screw that rotates by ~180o clockwise (on the extracellular side), ascending vertically by 6-8 A and changing its inclination angle from ~ 60o to ~35o. The amplitude of S4 vertical movements varies from ~0 A for S308 to ~14 A for S289. As this takes place, the S1, S2, and S3 helices rotate around S4 clockwise, conforming to earlier data [49] (*Fig. 6*).

Results of omega-current measurements [100] indicate that a salt bridge is formed between R1 in the S4 helix (R294 in Kv1.2) and E226 (in Kv2.1) in the S2 helix (*Fig. 6A*) in the closed channel state, stabilizing this state and preventing the penetration of ions from the extracellular aqueous antechamber to the inner one [53]. A substitution of R1 for a small non-polar amino acid causes the salt bridge’s destruction and formation of a through pore that allows protons to pass; this gives rise to the omega-current [100] confirmed by electrophysiological experiments [101, 102].

The obtained data indicate that Kv channels activation is linked to two basic types of conformational changes: (1) independent movement of VSD domains with a transition from the quiescent state to the “closed activated” state that keeps the pore domain gate closed [103-105]; and (2) cooperative transition of all VSD domains and the pore domain to the open state, when the pore domain gate is open for ion entry [104-106].

The role of gate-opening in the CMH model is attributed to the intracellular region of the S6 helix, while S5 initially rotates by ~7 A around the pore domain. Thus, the second basic rearrangement involves inclinations of the S4 helix that promote the inclination of the intracellular half of S5. This counterclockwise movement (on the extracellular membrane side) allows S4-S5 linkers and S6 helices in all four subunits to move together and to open the intracellular gate (*Fig. 6*).

The CMH models of closed and open channel demonstrate the following molecular details of the Kv channel activation mechanism (*Fig. 6*):

(1) The S4 helix moves vertically by ~6-8 A. The magnitude of S4 vertical movements in published structural models of the VSD domain transmembrane region in the open and/or closed states differs significantly: ~2–4 [48], ~3 [49] and 10–13 A [87, 100]. The model of the KvAP channel in the quiescent state that was published earlier [46, 81] suggested an amplitude of S4 vertical movements of ~15-20 A.

(2) The S3 helix moves relative to the S1, S2, and S4 helices. No significant movements of the S3 helix relative to all other segments of the VSD domain were noted in the previous publications.

(3) Coordinated movements of the S4 and S5 helices, the S4-S5 linker and S6 in all four subunits during the final opening of the channel. The mechanism of cooperative movements during channel opening had not been shown in any of the activation models published prior.

The CMH model conformity with the wide set of data that were previously considered as contradictory [47, 60, 72, 73, 93, 100, 107] allowed researchers to eliminate many contradictons in the discussion of the conformational rearrangements underlying the activation of Kv channels. Similar to the SHM model [108], the main movement in the CMH model is the axial rotation of S4 by ~180o. Like in the TM [48], the dielectric cavity contributes to the focusing of the transmembrane field, thus increasing the gate charge that links the VSD domain to the membrane potential energetically.

The CMH model was further improved using a full-atom molecular dynamics method in the membrane environment with an evident solvent [55, 109]. It was shown that the S4 α-helix spontaneously transits to the dexiotropic 3_10_ helix in the closed state of the Kv1.2 channel. This S4 helix conformation orients Arg towards the aqueous cavity in the VSD domain and allows salt bridge formation with the negatively charged amino acid residues along the S2 and S3 helices. The tendency of S4 to assume the 3_10_ helix conformation matches the crystal structures of the channels [44, 94] in which the inner part of S4 (~11 AA) forms the 3_10_ helix.

**Fig. 7:**
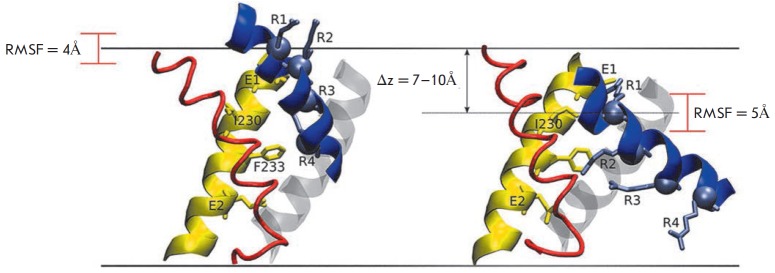
Comparison of Kv1.2 VSD domain models in the open (left) [21] and closed (right) conformations according to the consensus model (CM). The S1 helix is shown in grey; S2,in yellow; S3, in red; and S4, in blue. Cα atoms of the R294 residue move in the vertical direction by 7-10 A. The values of the root mean square fluctuations (RMSF) reflect the variation in the vertical *z *coordinate calculated for a Cα atom. The blue spheres with lateral radicals represent the basic charged amino acid residues of the S4 helix (R1–R4) that interact with amino acid residues in other helices (their side chains are marked) [55]

*(6) A consensus model (CM) *was developed by Vargas* et al*. [55] based on an improved CMH model [54]. They used data on the basic interactions between the amino acid residues of the VSD domain helices in the closed channel. Four key interactions (R294 and I177; R294 and I230; I230 and F267; F233 and R294 in Kv1.2 channel) were modeled using the MD method. The resulting four independent models were further averaged to create the CM of closed Kv1.2 conformation (*Fig. 7*).

The CM model conformed to all the experimental results that were used as the basis for the earlier models (SHM, TM, PM) [46, 84, 89, 110-113]. CM demonstrates that S4 moves in the vertical direction approximately by 7-10 A (*Fig. 7*) [55].

However, the CMH and the CM models cannot explain all the aspects of channel opening/closing. One of the reasons for that is the absence of information on intermediate Kv channel conformations. In this connection, attempts to determine the quantity of intermediate conformations and their structure by experimental methods and MM were made.

**Fig. 8:**
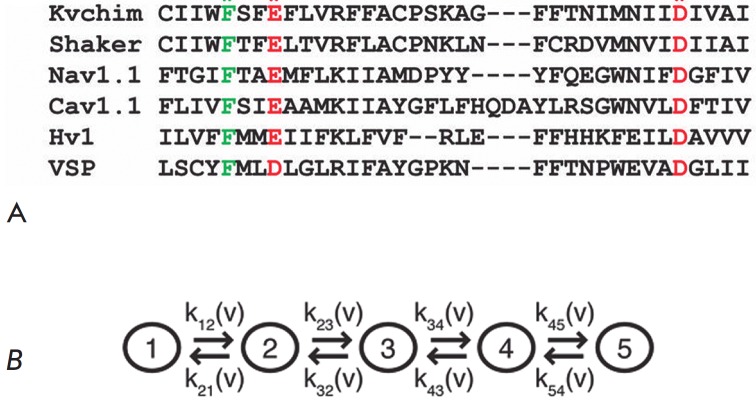
A. The charge transfer center (CTC) is highly conserved among VSD-containing proteins. The alignment of the sequences of the chimeric channel Kv1.2/2.1 (GI: 160877792), *Shaker *(GI: 13432103), human channel Nav1.1 (GI: 115583677), human channel Cav1.1 (GI: 110349767), human channel Hv1 (GI: 91992155), and VSP (GI: 76253898) is shown. Only CTC-forming portions of the S2 and S3 segments are given. The highly conserved residues forming the site are marked: F – green; E and D – red. F corresponds to Phe233 in the chimeric channel Kv1.2/2.1. B. The five-stage model of Kv channel activation with four steps of VSD movement. At each stage, different, positively charged residues of the S4 helix (R1– R5, indicated by numbers) consistently occupy the CTC (shown as a circle). When all four sensors reach stage 5, the pore opens [114]

MacKinnon *et al*. [114] found a highly conserved site in the VSD domain (*Fig. 8A*) formed by two negatively charged amino acid residues (D259, E236 – in *Shaker* channel) and one highly conserved (F233) that represented a “catalyst” for the transfer of each of the VSD domain basic amino acids (R1-R4, K5) through the membrane field.

**Fig. 9:**
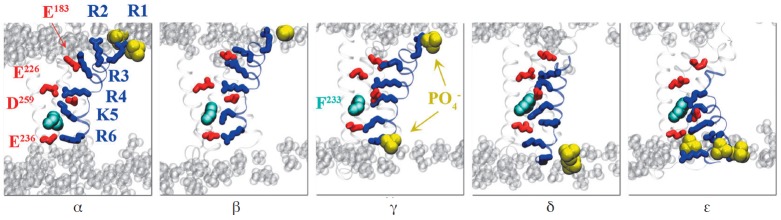
Five key intermediate stages of the Kv1.2 channel VSD domain, according to the model of charge transfer (MCT): initial upper position – α; three intermediate positions – β, γ, δ; and lower closed position – ε. The basic residues of the S4 helix are shown as blue sticks; amino acid residues and lipid PO4 - group that form salt bridges with R1–R5 are indicated by red sticks and yellow spheres, respectively. The highly conserved residue F233 of the S2 helix is shown as blue spheres [115]

This site is termed a charge transfer center (CTC ) [114]. During the S4 movement, each of its charged residues sequentially binds to this center; as a result, the whole activation/inactivation process divides into five consecutive stages (open channel, three intermediate stages, and closed channel), when the charged amino acids of the S4 helix (R1-R4, K5) sequentially bind to CTC (*Fig. 8B*). A group of French researchers [115] used the MD method and experimental data to study the VSD domain structure at different intermediate stages for a Kv1.2 channel placed on a lipid bilayer with an applied hyperpolarization potential. These five stages (states) are termed as follows: initial upper position, α; three intermediate positions, β, γ, δ; and lower closed position, ε (*Fig. 9*). During channel deactivation, the basic charged amino acid residues of S4 move from the external to the internal binding sites that represent the negatively charged residues of the S1-S3 segments (E183, E226, D259 and E236) as well as PO4 – groups of lipids. At the same time, the mass center of R1-R4 residues moves in the intracellular direction approximately by 12 A [78, 79]. Moreover, each of the four relocations is accompanied by the transition of one residue (K5, R4, R3 and R2) through the CTC area (*Fig. 9*). As a result,* (7) a model of charge transfer (MCT) *has been proposed.

In case process is observed from the external membrane side, the movement of S4 is accompanied by a slight inclination (~15°); S4 has a bigger inclination towards the membrane at the ε stage compared to the α stage. Earlier experiments [78, 79] demonstrated a moderate helical rotation of S4 clockwise (~45°) and a significant helical twisting counterclockwise (~90°) (*Fig. 9*). This model lacks significant (compared to other models) movement of the S4 helix until its top turns [50, 53, 96]. This model takes into account the data on the CTC presence [114]: the CTC site binds the basic residues K5, R4, R3, R2, and R1 in the conformations α, β, γ, δ, and ε, respectively. The CTC position in each conformation is preserved within the frames of the lipid bilayer central part [115].

**Fig. 10:**
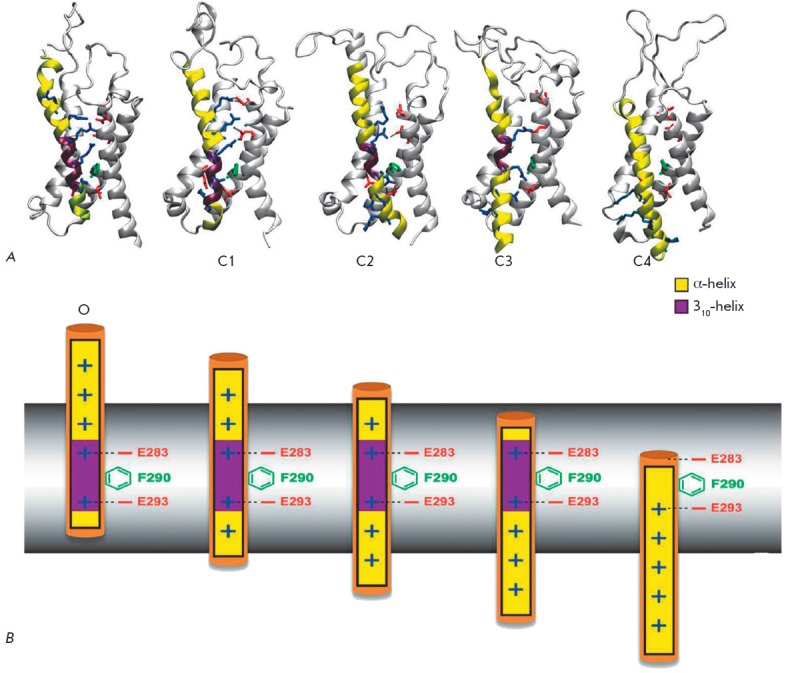
Model of Kv deactivation (MKD). Intermediate stages that the VSD domain of the *Shaker *channel passes during deactivation [116]. A. Molecular models of the VSD domain: O – open channel, C1-C2 – intermediate states, C3 – closed conformation, C4 – deep closed state, which occurs under special conditions. At each stage, the side chain of one of the Arg residues of the S4 helix (blue sticks) passes through the CTC (F290, green stick), while the Arg side chains located close to the CTC form salt bridges with the negatively charged residues of the S1-S3 helices (red sticks; E247 in S1 and E283 in S2 above F290; and E293 in S2 and 316D in S3 below F290). At all the stages, the portion of the S4 helix situated opposite F290 transits into a 3_10_ helix (purple), but at the C4 stage this portion is relaxed into an α-helix (yellow). Thus, the portion of the 3_10_ helix slides along the S4 segment without energy consumption, which prevents the rotation of this segment during the activation/deactivation of the channel. B. Schematic, demonstrating the movements of the S4 helix. Color coding as in *Fig. 10A*

The results of experiments on the creation of metal- ion (Cd^2+^) bridges later served as the basis for the identification of 20 new sites of interaction between the helices of the VSD domain [116]. These data were used for the modeling (by Rosetta method) of different intermediate conformations of the *Shaker *channel and for the creation of *(8) a model of Kv deactivation (MKD) *[116]. According to the MKD model, channel deactivation comprises five stages: O – open channel, C1-C2 – intermediate states, C3 – closed conformation, and C4 – deep closed state, which occurs under strong hyperpolarization (*Fig. 10*). The C3 stage corresponds to the CM of the closed conformation of the Kv channel [55].

In the MKD model, S4 moves fast in the intracellular direction during the deactivation, sliding along the S3 helix by at least 12 A (*Fig. 10*) [116]. As this takes place, the short region of the S4 helix (~10 aa) has the 310 helix conformation. At the open channel stage (O), the 310 helix is positioned in the middle of S4; as S4 moves down, the 310 helix moves along the S4 segment, always staying in the center of the membrane. The 310 part is limited by two out of five charged amino acids (R1-R4, K5) on either side, with its central part located opposite the CTC (F290). At the C4 stage, the last R1 residue passes below the hydrophobic lock formed by F290 and cannot form a salt bridge with E283; as a result, the structure of S4 relaxes into an α-helix. The C4 stage is difficult to achieve and is possible only under significant hyperpolarization [117]. The S4 segment must move by 17 Å in order to reach the C4 stage [116]. The existence of C4 is confirmed by experimental data [114, 118].

## ELECTROMECHANICAL COUPLING OF THE PORE AND VSD DOMAINS

It is still unknown how the VSD movement causes the opening of the channel pore; i.e., how the electromechanical coupling of VSD and the pore domains is implemented. It is known that the S4-S5 linker plays a major role in this process [31, 56], but the structural data are missing. In order to explain the functioning of the Kv channel and reveal the mechanism of electromechanical coupling of the pore and VSD domains, a group of researchers [56] studied the crystal structure of the open state of the Kv1.2/Kv2.1 channel [44, 119] using the MD method.

**Fig. 11:**
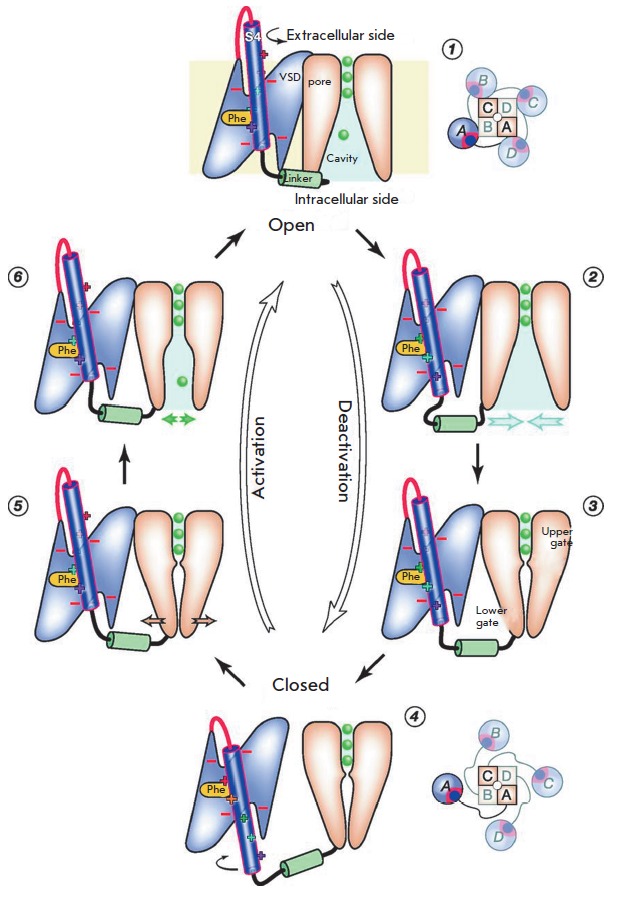
Mechanistic model of Kv activation/ deactivation (MMd) [56]. Effect of the hyperpolarization potential on the activated channel (1) initiates the inward movement of the S4 helix and weakens the bond between VSD and the pore domains. As a result, a depletion of ion transport in the pore cavity (2) and subsequent hydrophobic collapse of the pore occur. Closure of the upper (Ile402 in Kv1.2) and the lower gates [PVP motif; Leu331 (S5)–Pro405 (S6)] stops the ion current (3). The S4 helix continues to move inwardly; as soon as the S4 movement stops, the S4–S5 linker lowers completely and VSD domains are removed from the pore; the channel transits into the closed state (4). The impact of the depolarization potential on the closed channel leads to the movement of the S4 helix in an outward direction. The lower gate destabilizes when all four segments of the S4 and S4-S5 linker rise (5) and all VSD domains approach the pore again; the transition 4 => 5 represents the rate-limiting step of channel activation. Fluctuation of the lower gate causes opening of the pore and its partial rehydration. This allows potassium ions to enter into the pore and to initiate the channel conductance (6); the transition 5 => 6 is potential-independent. The presence of ions promotes complete rehydration of the pore leading to the complete opening of the upper and lower gates and returning the channel to the open state (1). Distribution of VSD domains (circles) relative to the pore domain (squares) is shown schematically (view from the extracellular side) [56]

An integral and detailed *(9) mechanistic model of Kv activation/deactivation (MMd) *(*Fig. 11*) was created. This model describes many previously unknown features of the process [56].

The channel deactivation causes a decrease in the ion transport that is accompanied by the exit of water from the hydrophobic cavity of the pore and a concurrent closing of the pore (pore collapse), explaining the osmotic dependence of the whole process of channel functioning [17, 18]. Next, the following steps take place: (1) full relaxation of VSD domains – movement of S4 inwards by ~15 A relative to the more stationary S1-S3a helices; (2) S4 rotation by ~120° that allows the charged amino acid residues to stay directed towards the VSD cavity; and (3) lateral detachment of VSD and the pore domains due to VSD rotation and movement outwards relative to the pore, allowing the pore to remain closed. During the activated state of the channel, the R4 residue is located in the membrane center, in a point of the peak transmembrane electric field, and serves as an initiator of movement for the gate charges. The CTC is the central hydrophobic residue F233 that divides the external and internal hydrated cavities of the VSD domain. The R2 and R3 residues move sequentially, while S4 movement inward usually stops when R1(Q) reaches F233. Several salt bridges are formed in the VSD domain, but S4 mainly interacts with the phosphate groups of the lipids. These data conform to those on the functional interaction of the VSD domain with lipids [13, 120].

The channel follows the same steps during the activation, but in the reverse order (*Fig. 11*): S4 swiftly moves outside by ~5-10 A. In the first step, the gate charges move fast because the majority of the salt bridges between S4 and the other segment of the VSD domain in the closed state are disrupted; these salt bridges are temporarily restored, while S4 moves outward, leading to the gradual slowdown of S4 movement. The VSD domains approach the characteristic state of an activated channel as soon as the S4 movement approaches its termination. A key difference as opposed to the deactivation is that all four VSD domains must be raised before the full opening of the channel pore; the channel with fully raised S4 segments breaks the packing of the S4-S5 linker together with S6 helix, allowing water and exiting ions to enter the pore again and restore the conductivity. The side chains of L331 (S5) and P405 (S6) move into a position that allows them to interact [17]. These rearrangements favor the binding of the S6 helix to the PVP motif, leading to the expansion at the intracellular side and full hydration of the pore. This is accompanied by the opening of the upper (hydrophobic) gate (I402) that allows the S5 site of the selectivity filter to fill up with K+ ions [121], and the channel transits to the fully open state. The S6 helix and S4-S5 linker adopt a closely packed configuration that stabilizes the pore opening.

We arrive at the conclusion that the opening and closing of the Kv channel is an energetically asymmetric process [56]. As far as the pore is more stable in the dehydrated closed state [17, 122] due to the hydrophobicity of its cavity [17], its closing does not require strong pressure by the S4 helix on the S4-S5 linker. On the contrary, channel activation requires the application of the depolarization work that stimulates the movement of the S4 helix through the membrane. Finally, S4 strongly pulls the S4-S5 linker, causing disruption of the S4-S5/S6 interaction and pore opening. Rather strong destabilization of the closed pore occurs only when all charged amino acid residues of the gate and S4-S5 linker are in the raised position. As a result, the fluctuations of the lower gate due to the disruption of linker interaction with the S6 helix allow partial (and then full) hydration of the pore cavity. The S4-S5 linker is strained during the activated channel state and relaxed in the quiescent state; this probably explains the conservatism of the linker length: a shorter linker inhibits channel closing, because S4 cannot move by a sufficient distance, while a longer linker inhibits the opening, because even complete outward relocation of S4 cannot efficiently pull the S6 helix using the S4-S5 linker [56].

Thus, the MMd model demonstrates that the S4- S5 linker and C-terminus of the S6 helix govern the process of channel opening/closing independently of the mechanism that raises and lowers the VSD domain [56]. The fact that it is possible to substitute the S3b-S4 paddle for a homologous sequence with the chimeric channel preserving the properties of the native channel [123, 124] indicates that this paddle is a key mechanic element in the process of channel activation/deactivation. The natural variability of the sequence of this functional region (S3b-S4 paddle and interacting region of the S4-S5 linker with the S6 helix) explains the differences in the activation parameters of Kv channels.

## CONCLUSIONS

A large number of models starting with the fundamental ones (SHM, PM and TM) and ending with modern ones (CMH, CM, MCT , MKD, MMd) [53, 54, 56, 115, 116] were proposed during the long history of studying the mechanism of Kv channels activation based on crystallography, mutational analysis, as well as MM and biophysical data. Such a synthesis of different methods and approaches allowed researchers to solve a very complicated problem: to identify the processes underlying the Kv channel activation without using the direct structural data on its closed conformation and intermediate states.

At present, researchers largely agree on a united model of Kv channel activation. The groups of scientists [53, 54, 56, 115, 116] has been able to obtain similar results, but the number of stages, the amplitudes of movements, and their directions still differ in different models. The estimation of the vertical translocation of S4 depends on how it is aligned relative to the open structure of the channel, as well as on the significant fluctuations due to the dynamic nature of the intermediate conformations [55].

A comparison of all available models of the VSD domains of the channel in the closed conformation [53, 54, 56, 115, 116] shows that all of them lay in a limit of ~3.5 A RMSD relative to the position of the Cα atoms [125]. Only one contradiction remains, which consists in determining the position of the R1 residue side chain. In some models [53, 55], this residue interacts with E226, while in others [56, 115] it interacts with D283. Each group of researchers asserts that their data are experimentally verified [50, 114, 118, 126]. It is possible that both conformations exist simultaneously in the case when a hyperpolarization potential is present [127].

The activation models consider two or three intermediate stages [53, 54, 56, 115, 116]. It should be noted that the intermediate conformations are unstable and hardly separable from each other [116]; therefore, different authors may well consider the same activation steps. Thus, in the MCT and MMd models [56, 115], three intermediate stages are considered, while in the MKD model [116] there are two stages, but all these models attempt to describe very similar processes. Probably, all models consider the same process, but they choose different intermediate points. Despite the slight differences in the models [53, 54, 56, 115, 116], all of them describe satisfactorily the generalized process of Kv channel activation and explain the basic principles of its functioning. The MMd model [56] is the most developed one. According to these principles, each VSD domain of the channel has deep aqueous cavities on both sides of the membrane, divided by a thin isthmus that contains a conserved Phe serving as a catalyst for moving the S4 gate charges. The positive basic amino acid residues of S4 are stabilized, interacting in pairs with the negative charges in the S1-S3 helices located along the S4 surface [49, 69, 71]. During activation, the positive charges “jump” from one negative charge to the next, leading to conformational change in the VSD. The S4 movement represents the combination of several processes: 1) inclination of the S4 helix in the membrane, 2) rotation on its axis, and 3) vertical and radial translocation. This movement displaces the S4-S5 linker and thus leads to the pore opening. The inner part of the S4 helix extends, while its two termini undergo screw-like rotation. The channel opening occurs after all VSD domains displace, while the channel closing requires displacement of only one of these domains. In order to reveal the precise mechanism of activation/ deactivation, especially the process of electromechanical coupling of the domains, it is necessary to elucidate the atomic structure of the Kv channel not only in the two final conformations (open and closed), but also in the intermediate states. This represents a very complicated problem, because these conditions are unstable and short-lived compared to the whole activation process timeframe.

## Abbreviations

Kv: voltage-gated potassium channel
aa: amino acid residue
VSD: voltage-sensing domain
SHM: sliding-helix model
TM: transport model
PM: paddle model
CMH: model of coordinated movement of helices
CM: consensus model
MCT: model of charge transfer
MKD: model of Kv deactivation
MMd: mechanistic model of Kv activation/deactivation
FRET: Förster resonance energy transfer
MM: molecular modeling
CTC: charge transfer center
MD: molecular dynamics
GC: gate channel
